# Evaluation of the Zucker Diabetic Fatty (ZDF) Rat as a Model for Human Disease Based on Urinary Peptidomic Profiles

**DOI:** 10.1371/journal.pone.0051334

**Published:** 2012-12-07

**Authors:** Justyna Siwy, Carlamaria Zoja, Julie Klein, Ariela Benigni, Wiliam Mullen, Bernd Mayer, Harald Mischak, Joachim Jankowski, Robert Stevens, Antonia Vlahou, Sophia Kossida, Paul Perco, Ferdinand H. Bahlmann

**Affiliations:** 1 Mosaiques Diagnostics, Hannover, Germany; 2 Charite- Universitaetsmedizin Berlin, Germany; 3 Mario Negri Institute for Pharmacological Research, Centro Anna Maria Astori, Bergamo, Italy; 4 School of Computer Science, University of Manchester, United Kingdom; 5 e BHF Glasgow Cardiovascular Research Centre, University of Glasgow, United Kingdom; 6 Emergentec Biodevelopment GmbH, Vienna, Austria; 7 Biomedical Research Foundation, Academy of Athens, Greece; 8 Department of Internal Medicine, Saarland University Hospital, Homburg, Germany; Baker IDI Heart and Diabetes Institute, Australia

## Abstract

Representative animal models for diabetes-associated vascular complications are extremely relevant in assessing potential therapeutic drugs. While several rodent models for type 2 diabetes (T2D) are available, their relevance in recapitulating renal and cardiovascular features of diabetes in man is not entirely clear. Here we evaluate at the molecular level the similarity between Zucker diabetic fatty (ZDF) rats, as a model of T2D-associated vascular complications, and human disease by urinary proteome analysis. Urine analysis of ZDF rats at early and late stages of disease compared to age- matched LEAN rats identified 180 peptides as potentially associated with diabetes complications. Overlaps with human chronic kidney disease (CKD) and cardiovascular disease (CVD) biomarkers were observed, corresponding to proteins marking kidney damage (eg albumin, alpha-1 antitrypsin) or related to disease development (collagen). Concordance in regulation of these peptides in rats versus humans was more pronounced in the CVD compared to the CKD panels. In addition, disease-associated predicted protease activities in ZDF rats showed higher similarities to the predicted activities in human CVD. Based on urinary peptidomic analysis, the ZDF rat model displays similarity to human CVD but might not be the most appropriate model to display human CKD on a molecular level.

## Introduction

Type 2 diabetes (T2D) is characterized by progressive failure of pancreatic insulin secretion to compensate for increased peripheral insulin resistance resulting in chronic hyperglycemia [1]. The prevalence of this common disorder has risen alarmingly in the past decade, in part due to increased obesity and sedentary lifestyle. Today, more than 346 million people worldwide are affected, and T2D is becoming an increasingly serious medical and - due to its costs - socio-economic issue [2].

Chronic hyperglycemia mostly affects the human vascular tree and promotes the development of micro- and macrovascular disease. About 30–40% of all patients develop microvascular disease, including retinopathy, and diabetic nephropathy (DN). Macrovascular disease in diabetic individuals affects the coronary, carotid and peripheral arteries, increasing the risk of myocardial infarction, stroke and diabetic foot disease [3]. The risk for cardiovascular (CV) events is 2- to 4- fold increased in diabetic patients as compared to patients without diabetes [4] and CV diseases (CVD) are responsible for up to 80% of premature excess morbidity and mortality in T2D. The development of these vascular complications is a complex and heterogeneous process, starting years before the onset of clinical symptoms. The ‘Cost of Diabetes in Europe - Type II study’ (CODE-2 study) that included data on 7000 people with T2D from eight European studies showed that 72% of the T2D patients had at least one complication and 24% had both, microvascular and macrovascular, complications [5].

We previously identified urinary biomarkers of CKD [6] and CVD [7] in different patient populations (diabetics and non-diabetics) using capillary electrophoresis coupled with mass spectrometry (CE/MS). Importantly, these urinary proteomic biomarkers have been validated and their association with CKD and DN (in the case of the CKD panel) and CVD (in the case of the CVD panel) was demonstrated in several large scale blinded studies [6]–[9].

Pre-clinical animal models are essential tools to gain a deeper understanding of the underlying pathophysiological changes leading to the development of T2D as well as its complications. Such models are mandatory for the development of therapeutic intervention for diabetes associated complications. Ideally, the animal models should display high similarity to the human disease on a molecular level.

To assess molecular similarity, we performed urinary proteome analysis in Zucker diabetic fatty (ZDF) rats on high fat diet, a recognized model of obesity, T2D, arterial hypertension and hyperlipidemia, that develop progressively diabetes-specific end-organ-damages [10], [11], and investigated the similarity between the human disease and the animal model on the molecular, urinary proteome level.

## Materials and Methods

### Animal Experiments

Male Zucker diabetic fatty (ZDF) rats (ZDF/Gmi-fa/fa; n = 20) and age matched LEAN control rats (ZDF/Gmi-fa/+; n = 17) were purchased from Charles River Laboratories Italia S.r.l., Calco, Italy, and housed in a constant temperature room with a 12-h dark/12-h light cycle. ZDF rats received high fat Purina 5008 diet (Charles River) to accelerate the development of the disease. Animal care and treatments were all conducted in conformity with the institutional guidelines that are in compliance with national (DecretoLegislativo n.116, GazzettaUfficialesuppl 40, 18 febbraio 1992, Circolare n.8, GazzettaUfficiale 14 luglio 1994) and international laws and policies (EEC Council Directive 86/609, OJL358-1, December 1987; Guide for the Care and Use of Laboratory Animals, US National Research Council, 1996). Animal studies were submitted to and approved by the Institutional Animal Care and Use Committee of Mario Negri Institute, Milan, Italy.

A group of ZDF (n = 10) and LEAN(n = 10) rats was studied at 2 months of age, the remaining ZDF (n = 10) and LEAN(n = 7) rats at 8 months of age. Blood glucose levels were assessed with an Ascensia glucometer (Bayer Diagnostics, Milan, Italy), serum cholesterol, triglycerides, and blood urea levels were measured by a Reflotron test (Roche Diagnostics, Indianapolis, USA). For the assessment of proteinuria, 24-hurine samples were collected using metabolic cages (Tecniplast, Varese, Italy)and proteinuria determined by the Coomassie method using a Cobas Mira plus autoanalyzer (Roche Diagnostic System, Basel, Switzerland). Serum and urinary creatinine were measured by Cobas Mira plus autoanalyzer (Roche Diagnostic System). Creatinine clearance rate was calculated as urinary creatinine X urine volume/serum creatinine and expressed as millilitres per minute. Systolic blood pressure (SBP) was measured in conscious rats with an occlusive tailcuffplethysmograph attached to a pneumatic pulse transducer with the BP-2000 BP analysis system (Visitech Systems, Apex, NC).

We obtained renal and cardiac tissue for morphologic and immunohistochemical analyses from the animals at 2 and 8 months of age. Kidneys were fixed overnight in Duboscq-Brazil, dehydrated in alcohol, and embedded in paraffin. Three-micrometer sections (Ultrotome V, LKB, Bromma, Sweden) were stained with hematoxylin and eosin and periodic acid-Schiff reagent. At least 100 glomeruli, including superficial and juxtaglomerullary cortical area, were examined for each animal. The extent of glomerular damage was expressed as the percentage of sclerotic glomeruli. Tubular damage in the tissue sections was evaluated as number of casts per high power field (HPF). Immunohistochemistry was performed on paraffin- embedded sections. Detection of monocyte/macrophage ED-1 surface antigen was performed by alkaline phosphatase- fast red technique using mouse monoclonal antibodies (1∶100, Chemicon, Temecula, USA). Positive cells were counted on an average of 30 randomly selected interstitial high- power fields (400x) for each animal. Type III collagen was detected by the ABC immunoperoxidase method using polyclonal rabbit anti-rat type III collagen antibody (1∶100, Chemicon). Visualization was performed by diaminobenzidine. The signal intensity was graded using the following scoring system: 0, no; 1, weak; 2, moderate; and 3, strong expression in at least 20 randomly selected high power fields (400x).

For cardiac histology, sections of the left ventricle (LV) were cut perpendicularly to its longitudinal axis and fixed in 4% neutral buffered formalin, for paraffin embedded sections.

### Sample Characteristic

Urine samples were collected from n = 10 ZDF and n = 10 LEANrats at 2 months of age and from n = 10 ZDF and n = 7 LEAN rats at 8 months of age. Collection was performed over a period of 24 hours in metabolic cages. Results for sample characteristic are expressed as mean ±SD. Data were analyzed using ANOVA coupled with Bonferroni post hoc analysis. Statistical significance level was defined as *P*<0.05.

### Sample Preparation

Immediately before preparation rat urine aliquots were thawed and 150 µl were mixed with 150 µl of 2 M urea, 10 mM NH_4_OH containing 0.02% SDS. Subsequently, samples were ultrafiltered using a Centristat 20 kDa cut-off centrifugal filter device (Satorius, Göttingen, Germany) to eliminate high molecular weight compounds. The obtained filtrate was desalted using a NAP5 gel filtration column (GE Healthcare Bio Sciences, Uppsala, Sweden) to remove urea and electrolytes. The sample was lyophilized in a Christ Speed-Vac RVC 2 18/Alpha 1 2 (Christ, Osterode am Harz, Germany) and stored at 4°C until use. Finally, shortly before CE/MS analysis, the samples were re-suspended in 10 µL HPLC-grade H2O. Samples were injected into CE-MS with 2 psi for 99 sec, resulting in injection volumes of ∼280 nL.

### CE-MS Analysis

CE-MS analysis was performed as described [12] using a P/ACE MDQ capillary electrophoresis system (Beckman Coulter, Fullerton, USA) on-line coupled to a MicroTOF MS (Bruker). The ESI sprayer (Agilent Technologies, Palo Alto, CA, USA) was grounded, and the ion spray interface potential was set –4.5 kV. Data acquisition and MS acquisition methods were automatically controlled by the CE via contact-close-relays. Spectra were accumulated every 3 s, over a range of m/z 350 to 3000.

**Figure 1:**
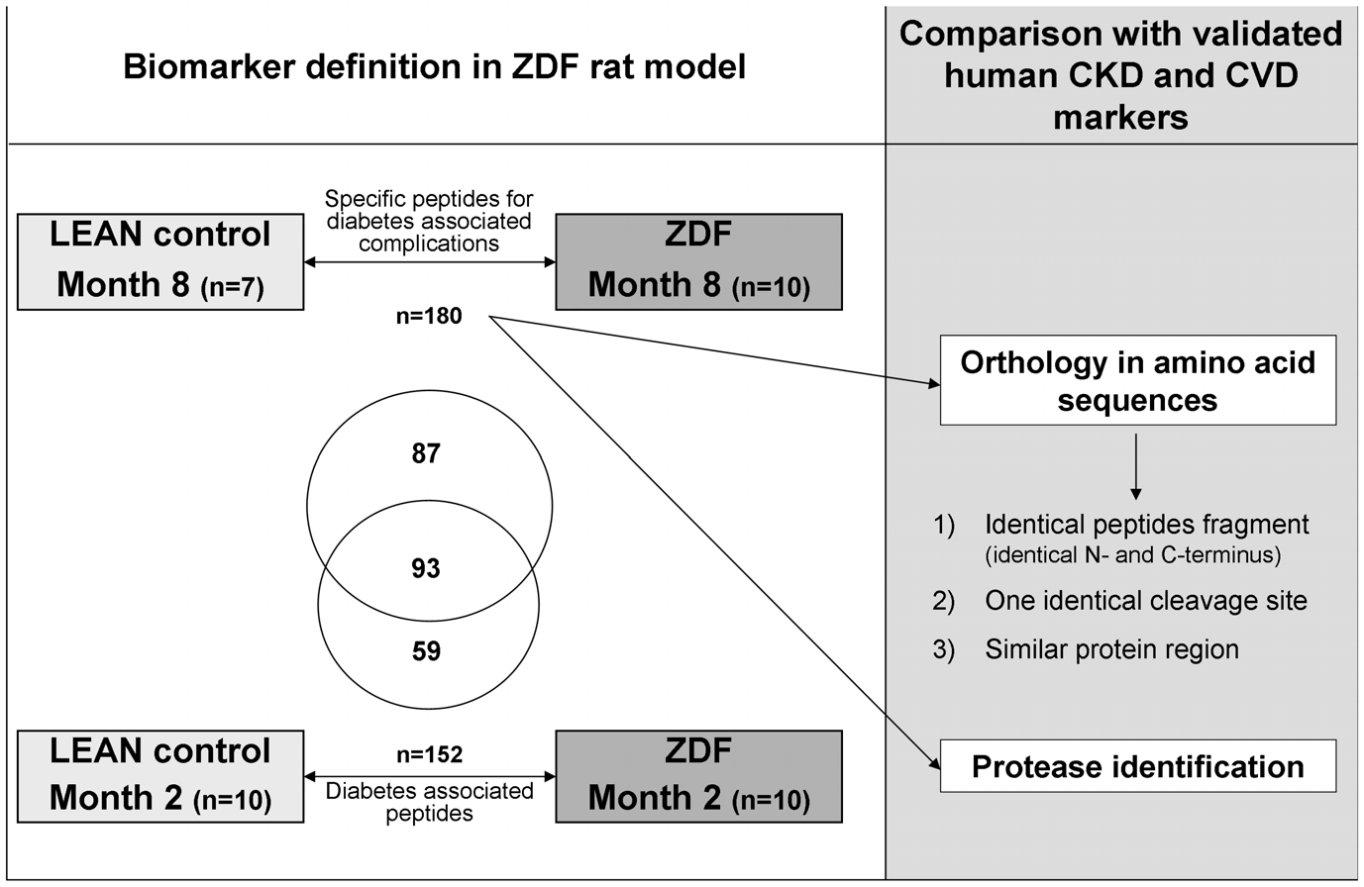
Study design. For biomarker definition urine samples of ZDF and LEAN control rats were used. The defined ZDF rat biomarkers at the late stage of disease (8 months) were compared based on the orthology of amino acid sequences, to the human CKD and CVD biomarkers. The overlap in sequenced urine peptide markers between early (2 months) and late stage disease ZDF rat markers is given in the Venn-diagram.

**Table 1:** Systemic, renal function and structure parameters measured in ZDF and LEAN rats at 2 and 8 months of age.

Groups	Body Weight (g)	Blood Glucose (mg/dl)	Serum Cholesterol (mg/dl)	Serum Triglycerides (mg/dl)	BUN (mg/dl)	Creatinine Clearance (ml/min)	Proteinuria (mg/24 h)	Glomeruli with sclerotic changes (%)	Tubular casts (n°/HPF)	Interstitial inflammation (ED1+cells/HPF)	Interstitial type III collagen (score)
**ZDF**											
2 months	324±16°°	200±64°°	120±5	497±166°°	24±3	3.56±0.32	75.79±25.08°	0	0	0.8±0.7	0
8 months	399±23* Δ	448±46** Δ	360±42** Δ	713±238**	39±8** Δ	1.20±0.12* Δ	379.74±84.96** Δ	18±6** Δ	5±2Δ	9.9±6.6** Δ	1.23±0.38Δ
**LEAN control**											
2 months	244±12	84±18	<100	77±6	19±3	3.06±0.32	16.92±0.88	0	0	0.5±0.6	0
8 months	429±17$	135±14	105±9	93±23	18±2	2.31±0.11	15.63±3.01	0	0	1.2±0.7	0.09±0.07

Values are expressed as mean ± SD.

°p<0.01 ZDF 2 months vs. LEAN 2 months;

°°p<0.01 ZDF 2 months vs. LEAN 2 months;

* p<0.01;

** p<0.001 ZDF 8 months vs. LEAN 8 months;

Δ p<0.001 ZDF 8months vs. ZDF 2months;

$ p<0.001 LEAN 8months vs. LEAN 2 months.

**Figure 2:**
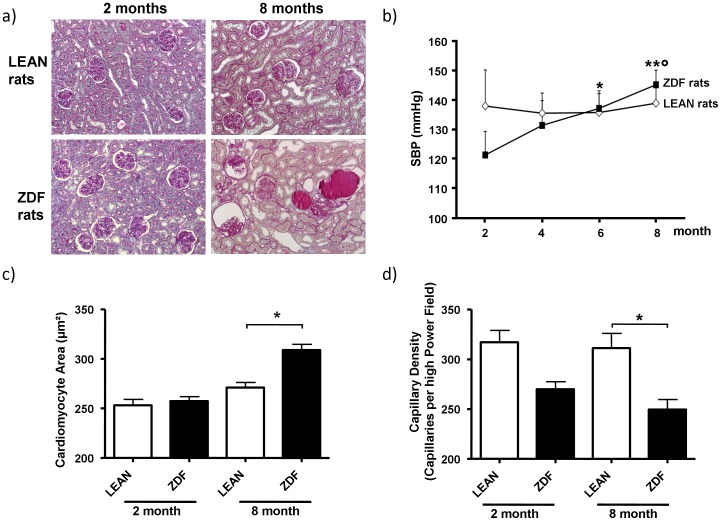
ZDF rats develop renal damage and cardiac structural changes after the onset of diabetes. A) Photomicrographs showing representative sections of kidneys from LEAN and ZDF rats at 2 and 8 months of age. No changes in renal morphology are observed in LEAN rats and in 2-month old ZDF rats. Sections from 8-month ZDF rats show glomerular sclerosis with thickening of the Bowman’s capsule and retraction of the tuft, tubular atrophy and dilation, and hyaline casts. PAS staining/200x; B) Time course of systolic blood pressure (SBP) in LEAN control and ZDF rats. C) Cardomyocyte area and D) Capillary density measured in cardiac tissue from LEAN and ZDF rats at 2 and 8 months of age. Values are mean±SD; *P<0.05; **P<0.01 vs. ZDF at 2 months; °P<0.05 vs. ZDF at 4 months.

**Figure 3:**
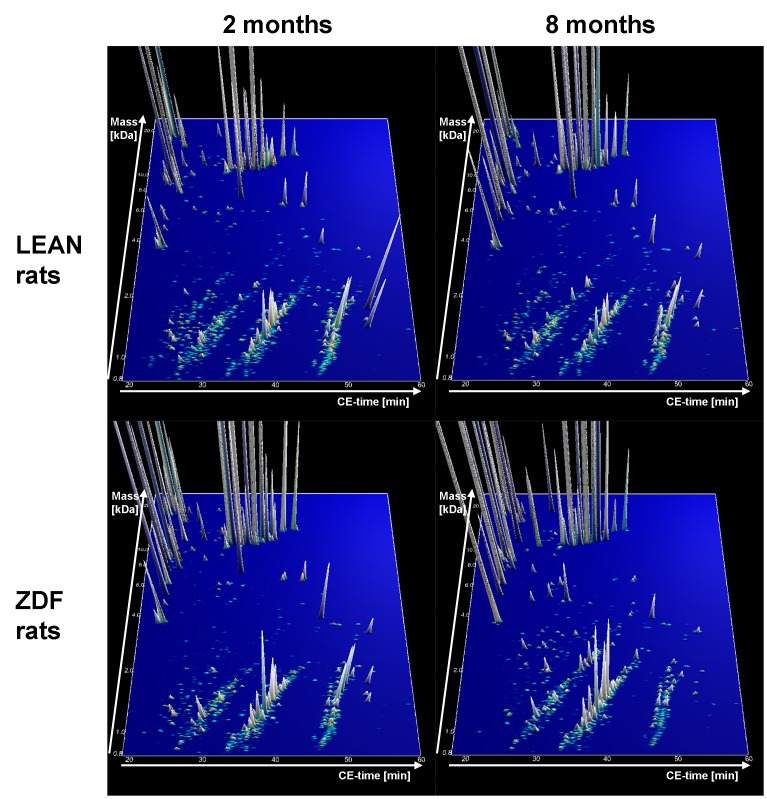
Group specific contour plots of LEAN control rats at 2 months (n = 10) and 8 months (n = 7) of age and type 2 diabetes ZDF rats at 2 and 8 months of age (n = 10 at each time). Each consisting of digitally compiled data sets of urine samples from all individual rats in a 3D depiction. Molecular mass of the analyzed polypeptides (0.8–25 kDa) in logarithmic scale is plotted against the CE migration time (18–60 min) with MS signal intensity in z-axis.

**Figure 4:**
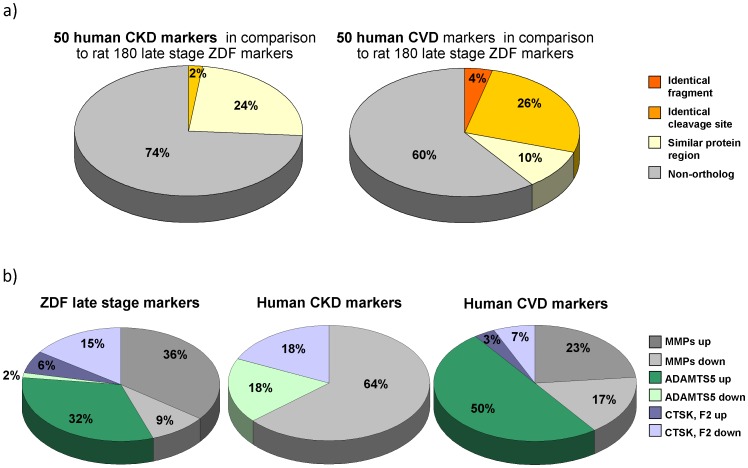
Similarity in amino acid sequences between rat and human biomarkers and predicted protease activities. a) Amino acid sequence orthology between the 50 most significant human biomarkers for CKD and CVD and the defined 180 rodent markers for diabetes associated complications. To examine the orthology between human and rat markers we applied three criteria: first, we looked for identical fragments (both cleavage sites identical in human and rat); secondly, we looked for fragments with one identical cleavage site and third, we looked for peptides from the same protein area with a minimum overlap in two amino acids. b) Predicted protease activities related to rat and human COL1A1 biomarker fragments. The relative number of specific cleavage sites for MMPs, ADAMTS5, CTSK and F2 and direction of amplitude regulation for COL1A1 markers in rat and human models are given.

**Table 2:** Identified specific cleavage sites for COL1A1.

		ZDF rat markers (8 months)		human CKD markers		human CVD markers	
specificcleavagesites (><)	Peptidase	up-regulated	down-regulated	up-regulated	down-regulated	up-regulated	down-regulated
PGK><QGA	MMP: 2, 3, 8, 12, 13	23	6	0	7	7	5
PQG><FQG	MMP: 2, 3, 9, 12						
PAG><ERG	MMP: 2, 9, 13						
PSG><FQG	MMP: 3, 8, 9, 12, 13						
PRG><LPG	MMP: 8, 9, 12, 13						
PAG><QPG	MMP: 8, 9*, 12, ADAMTS4						
PPG><KNG	MMP: 9, 13						
QPG><SPG	MMP: 9, 13						
PRG><ERG	MMP 9						
PAG><QQG	MMP: 8*, 9*, 12, 13						
PGP><SGK	ADAMTS5	21	1	0	2	15	0
PGP><AGP	ADAMTS5						
GPR><GPP	CTSK°, F2	4	10	0	2	2	1
PPQ><EKA	CTSK						

Three AA prior to and three AA after the observed cleavage point are indicated; the predicted protease (short name) and numbers on protease-to-urinary-peptide-pairs for COL1A1 based on the rat and human dataset are also provided. The trend of regulation (up- or down- in cases versus controls per species) is also shown.

°found only in CutDB-,

* found only in MEROPS-database.

### Data Processing and Cluster Analysis

Mass spectral ion peaks representing identical molecules at different charge states were deconvoluted into single masses using MosaiquesVisusoftware [13]. Migration time and ion signal intensity (amplitude) were normalized using internal polypeptide standards, as described elsewhere [12]. The resulting peak list characterizes each polypeptide by its molecular mass [Da], normalized migration time [min] and signal intensity. All detected polypeptides were deposited, matched, and annotated in a Microsoft SQL database. During initial clustering, polypeptides within different samples were considered identical, if the mass deviation was lower than ±50 ppm for masses <4.000 Da, for masses between 4.000 and 6.000 Da the 50 ppm entities gradually increasing to ±150 ppm and 150 ppm for features >6 kDa. Acceptable migration time deviation was<±1 minutes for 19 min, gradually increasing to<±2.5 min at 50 min.

### Statistical Analysis for Biomarker Definition

For the identification of potential biomarkers, the reported p-values were calculated using the Wicoxon Rank-Sum test followed by adjustment for multiple testing using the method described by Benjamini and Hochberg [14] Only proteins/peptides that were detected in a frequency of ≥70% in at least one of the diagnostic groups were considered for statistical analysis.

### Sequencing

The urine samples were analysed on a Dionex Ultimate 3000 RSLS nano flow system (Dionex, Camberly UK). The samples (5 µl) were loaded onto a Dionex 100 µm×2 cm 5 µm C18 nano trap column at a flowrate of 5 µl/min by a Ultimate 3000 RS autosampler (Dionex, Camberley UK) The composition of the loading solution was 0.1% formic acid and acetonitrile (98∶2). Once loaded onto the trap column the sample was then washed off into an Acclaim PepMap C18 nano column 75 µm×15 cm, 2 µm 100 Å at a flow rate of 0.3 µm/min. The trap and nano flow column were maintained at 35°C in a column oven in the Ultimate 3000 RSLC.

The eluent from the column was directed to a Proxeonnano spray ESI source (Thermo Fisher Hemel UK) operating in positive ion mode then into an OrbitrapVelos FTMS. The ionisation voltage was 2.5 kV and the capillary temperature was 200°C. The mass spectrometer was operated in MS/MS mode scanning from 380 to 2000 amu. The fragmentation method was HCD at 35% collision energy. The ions were selected for MS2 using a data dependant method with a repeat count of 1 and repeat and exclusion time of 15 s. Precursor ions with a charge state of 1 were rejected. The resolution of ions in MS1 was 60,000 and 7,500 for HCD MS2.

Data files from experiments performed on the HCD-enabled LTQ were searched against the IPI rat non-redundant database using the Open Mass Spectrometry Search Algorithm (OMSSA, http://pubchem.ncbi.nlm.nih.gov/omssa) and SEQUEST (by using Thermo Proteome Discoverer), without any enzyme specificity. No fixed modification was selected, and oxidation of methionine and proline were set as variable modifications. Mass error window of 10 ppm and 0.05 Da were allowed for MS and MS/MS, respectively. In the case of SEQUEST, the peptide data were extracted using high peptide confidence and top one peptide rank filters. The OMSSA results were further optimized by the use of the FDA Optimizer [15] 1% FDR was used as a cut-off value for reporting identified peptides. Accepted were peptides which were found with both search algorithms (OMSSA and SEQUEST), only with a mass deviation below ±80 ppm.

For further validation of obtained peptide identifications, the strict correlation between peptide charge at the working pH of 2 and CE-migration time was utilized to minimize false-positive identification rates [16]: Calculated CE-migration time of the sequence candidate based on its peptide sequence (number of basic amino acids) was compared to the experimental migration time. CE-migration time deviations below ±2 min corresponding to the CE-MS measurement were accepted.

### Protease Identification

A strict and a relaxed identification procedure was applied, to expand the number of potential protease involved in peptide generation. All peptide sequences were mapped to the full-length protein sequence and all complete cleavage sites: for peptide N-terminus, three amino acids from protein sequence before peptide start and three amino acids from peptide sequence after peptide start; for peptide C-terminus, three amino acids from peptide sequence before peptide end and three amino acids from protein sequence after peptide end. An in-house curated MEROPS/CutDB database (http://cutdb.burnham.org/) was used to search the six amino acids cleavage sites for matches to any of the protease cleavage sites in the database. As a next step, a relaxed search was conducted by mapping the cleavage sites to the database allowing up to 2 amino acids mismatches.

## Results and Discussion

To our knowledge, this study is the first to identify specific urinary peptides associated with diabetes-related complications in the ZDF rat model and to evaluate the similarity of this model to human urinary peptide markers for micro- and macrovascular complications. The study design is graphically depicted in **Figure 1**.

### Sample Characteristics

Characteristics for the sample cohort are given in **Table 1**. The ZDF rats were heavier than LEAN rats at 2 months of age. As in previous studies [10], the body weight of ZDF rats stabilized and was lower than that of LEAN rats at 8 months of age. Consistent with a diabetic phenotype, ZDF rats were hyperglycaemic, and dyslipidaemic compared with LEAN rats already at early (2 months of age) and more severely at late stage (8 months of age). Renal function was significantly impaired in ZDF rats at 8 months as indicated by increased BUN levels and reduced creatinine clearance. Proteinuria was already present at 2 months and progressively increased at 8 months in ZDF rats. No glomerular and tubulo-interstitial changes were observed at 2 months. By contrast, at 8 months in ZDF rats renal histology showed pathological changes, including glomerular sclerosis with thickening of the Bowman’s capsule and retraction of the tuft, tubular atrophy and dilation, and hyaline casts (**Table 1** **,** **Figure 2a**). Interstitial inflammation and accumulation of interstitial type III collagen were also observed (**Table 1**). In contrast, LEAN rats did not develop any histopathological changes. Blood pressure increased steadily in ZDF rats, while LEAN control rats had stable blood pressure during the experimental period (**Figure 2b**). ZDF rats showed an enlarged cardiomyocyte surface area compared to LEAN control animals at late stage of disease (**Figure 2c**). In addition, cardiac microvascular density was reduced in ZDF rats compared to LEAN control rats (**Figure 2d**) at both early and late stages of disease.

### CE/MS Analysis of Low Molecular Urinary Proteome Pattern in ZDF and LEAN Rats

From all urine samples high quality CE/MS data could be obtained. The compiled contour plots of early (2 months of age) and late (8 months of age) stage ZDF rats as well as LEAN control rats are displayed in **Figure 3**.

### Definition of Urinary Rat Late Stage Disease Markers and Comparison with Human Urinary Peptide Markers for Chronic Kidney (CKD) and Cardiovascular Disease (CVD)

For biomarker identification, we used urine samples from LEAN control (n = 7) and ZDF rats (n = 10) at month 8. We hypothesized that these markers should be associated with diabetes, diabetes-associated complications, or both. The comparison of the abundance of individual urinary peptides between cases and controls resulted in the identification of 531 peptides with significantly altered urinary excretion levels when adjusting for multiple testing (*P*-value <0.05). Sequencing information was obtained for 180 of the 531 marker candidates (**Figure 1**). The amino acid sequences, protein names, start and stop positions are all listed in **Table S1**. Two or more peptides originating from aminopeptidase N, apolipoprotein IV, collagen, contrapsin-like protease inhibitor, hemoglobin, osteopontin, proline-rich protein, seminal vesicle secretory protein, SMR1 and uromodulin were identified.

To assess similarity to human CKD and CVD, we compared the 180 rodent biomarkers of late stage disease with human urinary biomarkers of CKD (panel includes 273 sequenced biomarkers) [6] and CVD (panel includes 99 sequenced biomarkers) [7]. To focus on the most relevant disease-associated changes, the 50 most significant peptides (lowest *P*-values) in the CKD (**Table S2**) and CVD (**Table S3**) human panels were compared to the rat biomarkers. However, similar results (in terms of similarities in precursor proteins and trends of expression) were achieved when comparing all the peptides of each panel to the rat biomarkers (data not shown).

In the case of the CKD panel, this comparison revealed similarity in the precursor proteins of the rat and human biomarkers: In both species the biomarkers correspond to fragments of alpha-1-antytripsin (AAT), serum albumin (ALBU), collagen alpha-1(I) (COL1A1), collagen alpha-2(I) (COL1A2), collagen alpha-1(II) (COL2A1), collagen alpha-1(III) (COL3A1) and alpha-2-HS-glycoprotein (AHSG). We observed analogous regulation for fragments of AAT and ALBU (both increased in cases compared to controls) suggesting abnormal kidney filtration.

Plasma levels and activity of AAT are reported to be significantly decreased in diabetic patients [17]. Finding of increased levels of urinary AAT fragments [18] suggests increased clearance of AAT derived peptides in T2D paralleling albuminuria. Consistently, Varghese et al. identified urinary AAT as a candidate biomarker for glomerular disease [19] and Talmud and colleagues showed that AAT is associated with progression in T2D and that low levels of AAT promote atherogenesis [20].

Pathological changes associated with T2DN also include abnormal cross-linking of extracellular matrix compounds like collagen due to excessive presence of advanced glycemic end products (AGEs), resulting in abnormal matrix-matrix and matrix-cell interaction. These disturbances in collagen breakdown and chemical modifications of collagen contribute to atherosclerosis and fibrosis [21], [22].The modified secretion of many urinary collagen fragments may be reflective of these morphological observations. Thus, it is not surprising that in both species the largest number of peptides observed as specific for disease were COL1A1 fragments. However, the direction of the observed regulation for rodent and human CKD markers was not the same. In humans with CKD most COL1A1 fragments were decreased (14 out of 15) whereas nearly 60% were increased in urine of ZDF rats (37 out of 63), suggesting that, although displaying CKD features (**Table 1**), the ZDF rat might not “model” the molecular cascade promoting the changes towards renal failure in patients with CKD.

We next investigated whether we could find ortholog peptides between rat and the human CKD markers, assessing three levels of similarity (**Figure 1**): first, we queried the data for identical fragments (both cleavage sites identical in rat and human); secondly, we examined for fragments with one identical cleavage site. As the most relaxed requirement, we examined the data for peptides from the same protein area with overlap in a minimum of two amino acids (in all three criteria peptides were identified as ortholog when they showed the same regulation, increase or decrease in abundance, in both species). Surprisingly, we could not find convincing overlap of peptides found significantly altered in the ZDF model at 8 months with human urinary biomarkers for CKD. We could not detect any peptide that matched the first, most stringent criteria for comparison between the rat markers and the 50 human CKD markers. Two peptides were identified that had one identical cleavage site in rat and man, and further 12 could be identified that were from the same protein region and partly overlapping (**Figure 4a**).

Interestingly, many of the detected fragments in the rat model are also associated with progressive atherosclerosis. For example, AHSG, also known as fetuin-A [23], forms soluble complexes of otherwise insoluble calcium phosphate and thus acts as a potent inhibitor of pathological calcification. In addition, Fetuin-A or its variants seem to be involved in the progression of coronary atherosclerosis [24].

Next, to obtain information on the similarity of the rat ZDF model and human CVD, we also compared the identified markers in rat at late stage of disease with a set of previously reported human biomarkers for CVD [7] (**Table S3**). Similar precursor proteins of the biomarkers were present in rat and human samples, e.g.: AAT, COL1A1, COL1A2, COL2A1, and COL3A1. As in CKD, similar expression changes for fragments of AAT (increased in cases compared to controls in both species) were observed pointing towards a modification of the renal filtration barrier. In contrast however to CKD, most fragments of COL1A1 showed the same regulation in both species: In humans with CVD 18 out of 26 and in rats 37 out of 63 COL1A1 fragments were increased. Ortholog peptides of the CVD markers were identified as described above. Three rat markers were also found in the set of 50 highest significant human CVD markers ((SpGSpGPDGKTGPpGP,SpGSPGPDGKTGPpGP and ADGQpGAKGEPGDTGVKGDAGPpGP; the former in two variants modified by:proline and hydroxyproline, respectively). In addition, 13 fragments had one identical cleavage site and an additional 5 peptides were identified that showed an overlap in minimum of two AA (**Figure 4a**).

Collectively, the overlap between the human CVD biomarkers and defined biomarkers in the ZDF rats at late stage of disease was substantial, since several similar peptides could be identified that served as biomarkers in both cases, and with same trends of differential expression in cases versus controls. This is in line with data from Toblli et al. [25], indicating that ZDF rats may be an attractive experimental model to investigate cardiovascular damage related to human “metabolic syndrome”. Furthermore, Oltman et al. showed that vascular dysfunction in ZDF rats progress in coronary and mesenteric arteries and precedes changes in arteries [26]. We hypothesize that the changes observed are associated with, but generally not a result of changes in the glomerular filtration rate. The changes observed are most likely due to general change of abundance in both systemic changes (e.g. some of the collagen fragments observed) or due to functional changes in the kidney; it is reasonable to assume that to some extend increases in certain peptides may in part be a result of decreased tubular reabsorption [27], [28]. Tubular damage has a significant influence on the urinary proteome also in rats, and several of the biomarkers observed in this study were also found associated with tubular damage in rats, induced with cisplatin or gentamycin [12], [29]. Also the observed sclerotic glomeruli potentially contribute to urinary proteome changes.

However, we have no indication to assume that urinary proteases are significantly involved in the generation of the urinary peptides. This is based on the absence of significant changes in the urinary proteome after storage at room temperature for 6 h [30]. We thus hypothesize that changes in the urinary proteome are due to changes in protease activity in the tissue [31].

### In Silico Protease Identification

To obtain additional information on the underlying mechanism for similarity or differences of urinary biomarker peptides in rat and human, we expanded the analyses to the *in silico* identification of enzymes potentially involved in the generation of the urinary peptide fragments. The CutDB- [32] and MEROPS-database [33] were used for protease identification based on the known cleavage sites. We investigated the entire set of ZDF rat biomarkers and the 50 most significant human CKD and CVD biomarkers.

Since COL1A1 fregments are the predominant feature in ZDF rat late stage biomarkers, as well as human CKD and CVD biomarkers, we analysed in more detail the cleavage sites of all COL1A1 peptides to predict specific proteases involved in their generation. The N- and C-terminal cleavage sites for each fragment (6 AA, 3 before and 3 after the observed cleavage point) were analyzed with CutDB- and MEROPS-databases [32], [33]. We examined if either the cleavage site itself or a more relaxed pattern (i.e. no more than 2 unmatched AA) could be found.

In total, we identified 14 different specific cleavage sites (**Table 2**) for COL1A1. In **Figure 4** **b** the number of specific cleavage sites for matrix metallopeptidases (MMP, including MMP 2, 3, 8, 9, 12 and 13), ADAMTS5, cathepsin K (CTSK) and thrombin (F2) and trend of differential expression for COL1A1 markers in rat and human models are given. MMP is predicted to be increased in ZDF rats (23 cleavage sites of up- versus 6 of down regulated fragments), decreased in human CKD (0 vs. 7) and not clearly modified in human CVD (7 vs. 5). The increased abundance of these peptides indicative of MMP activity suggests an increase in MMP activity in this rat model. This observation is matched by observation in human CVD, where such peptides are also found increased. In contrast, peptides that likely result from MMP activity are reduced in human DN, indicating decreased MMP activity. The analysis of COL1A1 cleavage sites in ZDF rats also indicated increased activity of MMP. Increased activity of MMP13 has also been described in mouse atherosclerotic plaques [34].

The ADAMTS5 activity appears to be highly induced in ZDF rats (21 vs. 1) and human CVD (16 vs. 0) and possibly reduced in human CKD (0 vs. 2), based on COL1A1 fragments in ZDF rats and human CVD. Almost all fragments with a C-terminal PGP-motif are increased in rats with disease. The same regulation of fragments with PGP-motif was previously described by Delles et al. [7]. Furthermore, all COL1A1 cleavage sites with C-terminal GxPGP motifs, as reported in Delles et al. [7], were also found in ZDF rat as biomarkers for diabetes-associated complications. The increased activity of ADAMTS5 can explain the accumulation of fragments with C-terminal PGP-motif. Another possible explanation could be decreased activity of prolyloligopeptidase which cleaves specific GxPGP-motifs. Decreased activity would result in accumulation of the PGP-containing substrate peptides. However, such a mechanism would require a concomitant decrease of the expected reaction products. As this was not observed, decreased prolyloligopeptidase activity does not appear to be the cause for the increase in PGP-containing peptides.

The CTSK/F2 activity seems to be decreased in all pathologies. Albeit based on a small number of observations, we find reduction of activity in ZDF rats (4 vs. 10), as well as in human CKD (0 vs. 2) and CVD (2 vs. 1).

As one of the most interesting findings is the presence of the three identical peptides in the rat and CVD panels, two of which also appear at very early stage of the disease in the former (described below). The three ZDF rat markers identical to the human CVD-specific COL1A1 fragments are all C-terminal cleavage sites specific for ADAMTS5 and all N-terminus cleavage sites are specific for MMP9 or MMP13. These fragments also include the specific C-terminal PGP motif leading to the hypothesis that increased activity of ADAMTS5 may be related to molecular mechanisms of disease early development. This hypotheses based on the cross-species molecular evaluation presented herein may open new directions in the investigation of early development of diabetic complications, and apparently merit further experimental verification.

### Definition of Urinary Rat Early Stage Disease Markers

We also investigated if proteomic differences could also be observed in ZDF rats at 2 months of age where no morphological signs of nephropathy were present. The group of LEAN controls (n = 10) was compared to ZDF(n = 10) rat samples at the early stage of disease (2 months of age). Five hundred forty two significant peptides were defined and 152 of them could be sequenced (**Figure 1** **, Table S1**). Similar to the late stage markers, at least two fragments of aminopeptidase N, apolipoprotein IV, collagen, contrapsin-like protease inhibitor, prolin-rich protein, seminal vesicle secretory protein and uromodulinwere identified. In addition, two fragments of alpha-1-aminopeptidase and complement C3 were identified.

Some of the identified biomarkers in ZDF rats at the early stage of disease may not only be specific for diabetes, but could also be biomarkers specific for early-stage diabetes associated complications. Indeed, 93 of these markers (61%) overlap with the 180 markers defined at the late stage of disease (**Table S1**). Interestingly, in this set of overlapping peptides two of the three identical markers to the human CVD markers were included (SpGSpGPDGKTGPpGP, SpGSPGPDGKTGPpGP).

### Conclusions

Molecular features are key elements in pathology and in general the target structure for pharmacological intervention. Among those, proteins appear especially relevant. Consequently, changes on the proteomic level reflect the molecular pathology. Based on these considerations, we aimed at comparing proteomic changes in a widely used animal model to those observed in man.

The present study demonstrates the potential of (urinary) proteome analysis for the assessment of molecular similarity of animal models with human disease. Such assessment of similarity appears to be one of the most important issues in the development and application of animal models for human disease. It is expected that the approach presented here will open new avenues in the establishment of appropriate animals models, and increase the portability of results obtained in animal models towards human disease. The data also emphasize the complex nature of diabetic end–organ damages and the need for additional animal models with features more analogues of those seen in human CKD. The findings suggest a re-evaluation of the ZDF model. Based on our results, the ZDF rat may be more suitable to study the macrovascular branch within the pathophysiologic cascade of diabetic angiopathies (i.e CVD), but does not appear to represent an appropriate model for microvascular disease (i.e. CKD).

## Supporting Information

Table S1 **Table listing 180 identified ZDF rat peptides specific for diabetes associated complications at late stage of disease and the 152identified ZDF rat diabetes-specific peptides at early stage of disease.** Shown are sequences (modified amino acids: p = hydroxyproline; k = hydroxylysine; m = oxidized methionine), protein names, start and stop amino acid.In addition, statistical data for late and early stage samples:P-values (adjusted using Benjamini-Hochberg), mean signal intensity (amplitude) in the two groups of the cohort, and the regulation in ZDF compared to controls. The orthology of the late stage ZDF markers to the high significant human CKD and CVD is also given. The 93 overlap markers between the early and late stage of disease are marked in bold.
(XLS)

Table S2 **Listed are 50 of 273 previous defined human CKD markers** [**6**] **with lowest P-values.** Shown are sequences (modified amino acids: p = hydroxyproline; k = hydroxylysine; m = oxidized methionine), protein names, start and stop amino acid.In addition, adjusted P-values in diabetes cohort; mean signal intensity in DNgroup which contained 313samplesfrom diabetic patients with CKD and in controlgroup contained 512samplesfrom diabetic patients without CKD and the regulation in cases compared to controls. The orthology to the rat markers is also given.
(XLS)

Table S3 **Listed are 50 of the 99 sequenced of 238 previous defined CVDmarkers** [**7**] **with lowest P-valuesShown are sequences (modified amino acids: p = hydroxyproline; k = hydroxylysine; m = oxidized methionine), protein names, start and stop amino acid.** In addition, adjusted P-values in diabetes cohort; mean signal intensity in DN group which contained 313samplesfrom diabetic patients with CVD and in control group contained 512samples from diabetic patients without CVD and the regulation in cases compared to controls. The orthology to the rat markers is also given.
(XLS)
